# Neuroimmunity dynamics and the development of therapeutic strategies for amyotrophic lateral sclerosis

**DOI:** 10.3389/fncel.2013.00214

**Published:** 2013-11-19

**Authors:** Melissa Bowerman, Thierry Vincent, Frédérique Scamps, Florence E. Perrin, William Camu, Cédric Raoul

**Affiliations:** ^1^The Neuroscience Institute of Montpellier, INM, INSERM UMR1051, Saint Eloi HospitalMontpellier, France; ^2^Department of Immunology, Saint Eloi HospitalMontpellier, France; ^3^Integrative Biology of Neuroregeneration, Faculty of Science, University of Montpellier 2Montpellier, France; ^4^Department of Neurology, ALS Reference Center, Gui-de-Chauliac HospitalMontpellier, France

**Keywords:** inflammation, microglia, lymphocytes, astrocytes, hyperexcitability, cytokine, therapy

## Abstract

Amyotrophic lateral sclerosis (ALS) is a fatal paralytic disorder characterized by the progressive and selective loss of both upper and lower motoneurons. The neurodegenerative process is accompanied by a sustained inflammation in the brain and spinal cord. The neuron-immune interaction, implicating resident microglia of the central nervous system and blood-derived immune cells, is highly dynamic over the course of the disease. Here, we discuss the timely controlled neuroprotective and neurotoxic cues that are provided by the immune environment of motoneurons and their potential therapeutic applications for ALS.

## INTRODUCTION

Amyotrophic lateral sclerosis (ALS) is a neurodegenerative disease characterized by the selective and progressive loss of upper and lower motoneurons, with both genetic and sporadic events contributing to the development of the pathological process (reviewed in Bento-Abreu et al., 2010). The term “neuroinflammation” has been attributed to the inflammatory response that occurs within the central nervous system (CNS) concomitantly to neurodegeneration (reviewed in Glass et al., 2010). Astrocytes, microglia, and immune cells are the key cellular modulators of neuroinflammation and have all been shown to actively participate in ALS pathogenesis (Glass et al., 2010; McCombe and Henderson, 2011; Philips and Robberecht, 2011). Importantly, recent reports have highlighted the presence of both neuroprotective and neurotoxic inflammatory cells in ALS animal models and patients that appear to be mainly dependent on the stage of disease progression. Seeing as reviews on the relationship between astrocytic activation and ALS are numerous, we will focus herein on the dynamic functional changes of microglia and immune cells that take place during ALS pathogenesis. A better understanding of these time-dependent modifications is of utmost importance for the development of ALS therapeutic strategies aimed at targeting the neuroinflammatory process.

## A ROLE FOR MICROGLIA IN NEUROINFLAMMATION

### ACTIVATION PROFILE IN HUMAN AND ANIMAL MODELS OF ALS

Microglia, the resident immune cells of the CNS, constantly survey the environment and become activated upon alterations resulting from disease or injury eliciting a strong pro-inflammatory response (reviewed in Hanisch and Kettenmann, 2007). In ALS patients, reactive microglia are observed in the motor cortex, motor nuclei of the brainstem, the entire corticospinal tract, the spinal cord, and within the cerebrospinal fluid (CSF; Engelhardt and Appel, 1990; Kawamata et al., 1992; Banati et al., 1995). Given the relationship between astrocytes and microglia and the importance of astrocytosis in ALS (Davalos et al., 2005; Yamanaka et al., 2008), it has been hypothesized that microgliosis may also participate in ALS pathogenesis.

In rodent ALS models, microgliosis occurs in pre-symptomatic and symptomatic *SOD1*^*G93A*^ mice (Hall et al., 1998; Alexianu et al., 2001; Petrik et al., 2007; Gerber et al., 2012) and at both onset and early-stage of the disease in *SOD1*^*G37R*^ mice (Boillee et al., 2006). An in-depth *in vivo* characterization of microgliosis in *SOD1*^*G93A*^ mice shows that microglia are highly reactive in pre-symptomatic stages while they lose their ability to monitor the environment as the disease progresses (Dibaj et al., 2011). Indeed, microglia isolated from either neonatal or early onset *SOD1*^*G93A*^ mice display an activated M2 phenotype and enhance motoneuron survival while microglia isolated from either adult or end stage mice have a classically activated M1 phenotype and induce motoneuron death (Weydt et al., 2004; Liao et al., 2012). In the pre-symptomatic and symptomatic *SOD1*^*G93A*^ rat model, microglia aggregates are detected in both the spinal cord and brainstem and display a degenerative and apoptotic phenotype at end stage (Fendrick et al., 2007; Graber et al., 2010). Moreover, microglia of pre-symptomatic *SOD1*^*H46R*^ rats express the proliferating marker Ki67 and the phagocytic markers ED1 and major histocompatibility complex (MHC) class II (Sanagi et al., 2010; Bataveljic et al., 2011). These data suggest that microgliosis not only typifies ALS but that microglia function changes during disease progression, thus exerting differential effects on motoneurons.

### A ROLE FOR MICROGLIA IN ALS PATHOGENESIS

A key finding supporting the contribution of microglia in ALS pathogenesis is the significant extension in lifespan and delay in disease progression when the mutant protein is specifically deleted from macrophages and microglial lineages in both *SOD1*^*G37R*^ and *SOD1*^*G85R*^ mice (Boillee et al., 2006; Wang et al., 2009). Similarly, bone marrow transplantation (resulting in donor-derived microglia) of *SOD1*^*G93A*^ microglia into *PU.1*^-/-^ mice (that lack CNS microglia at birth) did not induce neurodegeneration whereas wild-type donor-derived microglia transplantation into *SOD1*^*G93A*^; *PU.1*^-/-^mice improved survival (Beers et al., 2006).

However, phenotypical analysis of microglia in different regions of *SOD1*^*G93A*^ spinal cord suggests that both neuroprotective and neurotoxic population of microglial cells may co-exist during the disease and that depletion of proliferative microglia does not prevent motoneuron degeneration (Gowing et al., 2008; Beers et al., 2011b). Together, these studies thus suggest that microglia participates, through a complex balance between neuroprotective and neurotoxic signals, to ALS disease progression.

### PROPOSED MECHANISMS OF MICROGLIAL-DERIVED NEUROTOXICITY

Various misregulated pathways within ALS microglia have been identified that may influence motoneuron survival. Endoplasmic reticulum (ER) stress is a characteristic of ALS pathogenesis (reviewed in Lautenschlaeger et al., 2012). In microglia of both sporadic ALS patients and symptomatic *SOD1*^*G93A*^ mice, there is an increased expression of C/EBP homologous protein (CHOP; Ito et al., 2009), a member of the apoptotic ER stress pathway (reviewed in Oyadomari and Mori, 2004). It remains unclear if it directly participates in microglial neurotoxicity but exposure of microglia to interferon gamma (IFNγ), which levels are increased in the spinal cord of ALS mice and patients (Aebischer et al., 2011; Aebischer et al., 2012), elicits inducible nitric oxide (NO) synthase (iNOS) expression. The subsequent production of NO can cause an ER stress response that involves CHOP (Kawahara et al., 2001). Interestingly, several SOD1 mouse models show initiation of a specific ER stress response accompanied by microglial activation (Saxena et al., 2009).

Activation of the ligand-dependent CD14 lipopolysaccharide (LPS) receptor located at the microglial surface (Lacroix et al., 1998) initiates a pro-inflammatory Toll-like receptors (TLRs) dependent cascade (Laflamme and Rivest, 2001; Laflamme et al., 2001). Importantly, neurotoxic microglia activation by extracellular SOD1^*G93A*^ is mediated by the CD14-TLR2 pathway and induces a subsequent release of pro-inflammatory cytokines, including tumor necrosis factor alpha (TNFα) and interleukin (IL)-1β (Liu et al., 2009; Zhao et al., 2010). Moreover, microglia from sporadic ALS patients show an enhanced TLR2 immunoreactivity (Casula et al., 2011). Microglia may thus participate in motoneuron loss following the specific activation of the CD14-TLR pathway by secreted SOD1 mutant, therefore propagating pro-inflammatory stimuli.

The release of extracellular nucleoside di- and tri-phosphates, in particular ATP, by degenerating neurons can elicit microglia activation through the ionotropic P2X and metabotropic P2Y purinergic receptors which can subsequently elicit a pro-inflammatory response, chemotaxis, and phagocytosis (reviewed in Inoue, 2006; Bours et al., 2011). Notably, P2X is increased within spinal cord microglia of ALS patients (Yiangou et al., 2006). Embryonic microglia and neonatal primary microglial cultures from mutant SOD1 mice display an upregulation of P2X_4_, P2X_7_, and P2Y_6_ receptors (D’Ambrosi et al., 2009). Further, activation of P2X_7_ in *SOD1*^*G93A*^ microglia leads to the production of significantly higher levels of TNFα, which has a neurotoxic effect on motoneuron cultures (Ugolini et al., 2003), and of cyclooxygenase-2 (COX-2), which produces the potent inflammatory mediators prostaglandins (D’Ambrosi et al., 2009).

Moreover, a reduced ATP hydrolysis activity in mutant SOD1 microglia, suggests a potentiation of a purinergic-mediated inflammation that can participate to the neuroinflammatory state of microglial cells. Since ATP induces an astrocytic neurotoxic phenotype through P2X_7_ receptor signaling (Gandelman et al., 2010), one can hypothesize that increased extracellular ATP in ALS, whether exacerbated by motoneurons and/or microglia contributes to the pathogenic microgliosis.

### THE POTENTIAL INFLUENCE OF MICROGLIA ON NEURONAL EXCITABILITY

There is presently few assessment of the influence of microglia on motoneuron electrophysiology. However, studies on peripheral nerve or spinal cord injuries show that microglia activation has prominent effects on neuronal inhibitory control and loss of inhibitory control is a contributing mechanism to the motoneuron hyperexcitability that typifies ALS pathogenesis in humans (Bae et al., 2013).

Loss of neuronal inhibitory control occurs by several means including decrease in gamma-aminobutyric acid (GABA)ergic interneurons combined with changes in the expression of the GABA_A_ receptor messenger RNA subunit (Petri et al., 2003; Maekawa et al., 2004). GABA_A_ and glycine receptors are chloride (Cl^-^) channels and the expression of cation-chloride co-transporter contributes to inhibitory effects of these Cl^-^ currents (Blaesse et al., 2009). Indeed, the entry of Cl^-^ following the opening of GABA_A_ and glycine receptor-gated Cl^-^ channels inhibits neuron excitability by hyperpolarizing membrane potential. Under physiological condition, low intracellular Cl^-^ concentration [Cl^-^]_i_ is maintained by the potassium (K^+^)-chloride co-transporter KCC2 that extrudes Cl^-^ from mature neurons (Rivera et al., 1999). Stimulation of spinal microglia following peripheral nerve injury induces a decrease in KCC2 expression among dorsal horn nociceptive neurons (Coull et al., 2003). KCC2 decrease is induced by the brain-derived neurotrophic factor (BDNF) and this is consistent with the previous observation that BDNF can be produced by non-neuronal cells involved in immune responses, including T and B lymphocytes, monocytes, and microglia (Kerschensteiner et al., 1999; Coull et al., 2005). BDNF produces a depolarizing shift in the anion reversal potential of dorsal horn lamina I neurons due to an increase in [Cl^-^]_i_. This shift prompts an inversion of inhibitory GABA currents that contributes to neuropathic pain following nerve injury (Coull et al., 2005). Decrease in KCC2 expression is thus responsible for the excitatory effects of GABA on neurons. Microglia activation and BDNF secretion are mediated through ATP activation of microglial P2X receptors. As discussed earlier, P2X receptors might be involved in ALS pathology since a higher density of P2X_7_-immunoreactive microglial cells/macrophages are found in affected regions of spinal cords from ALS patients (Yiangou et al., 2006). Furthermore, levels of BDNF have been found to be increased in microglial cells isolated from ALS mice at the onset of disease and KCC2 is decreased in vulnerable motoneurons in *SOD1*^*G93A*^ mice (Fuchs et al., 2010; Liao et al., 2012). Additionally, BDNF might play a role in the influence of microglia on motoneuron electric activity as suggested by work on spasticity. Spasticity is characterized by a velocity-dependent increase in muscle tone resulting from hyperexcitable stretch reflexes, spasms and hypersensitivity to normally innocuous sensory stimulations. Spasticity develops following spinal cord injury and is also regarded as an ALS clinical symptom (Rowland and Shneider, 2001). The main mechanism hypothesized to be responsible for spasticity is increased motoneuron excitability and increased synaptic inputs in response to muscle stretch due to reduced inhibitory mechanisms. Recently, it has been demonstrated that, following spinal cord injury, increased levels of BDNF mediated spasticity, due to post-transcriptional downregulation of KCC2 (Boulenguez et al., 2010). Together, these studies suggest that reactive microglia in ALS may exert an aberrant effect on the electrical activity of motoneurons and highlight the importance of furthering our understanding of this functional interaction.

Lastly, a hypothetical scenario relates to the defect in astrocytic glutamate transporter and the neurotoxic accumulation of the excitatory amino acid. It has been demonstrated that TNFα promotes glutamate release by activated microglia through the cystine/glutamate exchanger (Xc; Piani and Fontana, 1994). Though the implication of the Xc system in ALS has not yet been investigated, it may represent a potential mechanism of microglia-mediated excitotoxicity that warrants further study (Qin et al., 2006).

## THE DUAL ROLE OF NEUROIMMUNITY IN MOTONEURON DISEASE

### PATHOLOGICAL PHENOTYPE OF THE IMMUNE SYSTEM IN ALS

In addition to astrocytes and microglia, blood-derived immune cells may also play synergistic and critical functions during disease progression. Presence of a systemic immune activation is suggested by abnormalities observed in the blood and the CSF of ALS patients such as increased numbers of circulating lymphocytes (CD4^+^ helper T cells, CD8^+^ cytotoxic T lymphocytes, CTL, and natural killer, NK cells), increased expression of MHC class II molecules on monocytes as well as higher levels of inflammatory chemokines and cytokines (regulated on activation normal T cell expressed and secreted, RANTES, monocyte chemotactic protein, MCP-1, IL-12, IL-15, IL-17, and IL-23; Zhang et al., 2005; Rentzos et al., 2007, 2010, 2012; McCombe and Henderson, 2011). Further, post-mortem studies of brain and spinal cord lesions from ALS patients show that the activation and proliferation of microglia is associated with an infiltration of activated macrophages, mast cells and T lymphocytes which are found in close proximity to degenerating tissues (Engelhardt et al., 1993; Graves et al., 2004; Lewis et al., 2012). An in-depth autopsy of six ALS patients reveals an enrichment of T-cell receptor Vβ2-positive T cells in the spinal cord and CSF, suggesting an antigen-driven T cell selection (Panzara et al., 1999). Finally, ALS patients with a more rapidly progressing pathology show decreased numbers of regulatory T lymphocytes (Tregs), suggesting that the number of Tregs is inversely correlated with disease progression (Beers et al., 2011a; Rentzos et al., 2012). Tregs secrete anti-inflammatory cytokines such as IL-4, IL-10 and transforming growth factor beta (TGF-β) and has been show to induce the production of the neurotrophic factors glial-derived neurotrophic factor (GDNF) and BDNF by astrocytes (Reynolds et al., 2007). Tregs are also able to dampen a T helper (Th)1 pro-inflammatory response and attenuate toxic microglial responses. Contribution of the innate immune system is also suggested by the presence of immunoglobulins and complement deposition as well as a significant increase of NK cells in the blood of ALS patients (Donnenfeld et al., 1984; Engelhardt and Appel, 1990; Rentzos et al., 2012). While these investigations of ALS samples and tissues do not assess the contributory role of the immune system to disease pathogenesis, they do highlight its active presence.

In support of what is observed in humans, ALS rodent models also display a particular immunological phenotype. Indeed, *SOD1*^*G93A*^ mice have allowed the demonstration that the inflammatory cellular subtypes are phenotypicaly and functionally different depending upon the disease stage (Liao et al., 2012). During the initial stages, infiltrating CD4^+^ T cells are mainly Th2 (IL-4^+^) while there is a skew toward Th1 (IFNγ^+^) cells and CD8^+^ T cells (both IL-17A positive and negative) as the disease progresses (Fiala et al., 2010; Beers et al., 2011b). Alteration in inflammatory cell subtypes is associated with, and maybe driven by, differences in Tregs. Interestingly, early symptomatic *SOD1*^*G93A*^ mice have an increased number of Tregs and a decreased proliferation of effectors T lymphocytes (Teffs), whereas a decreased numbers of Tregs and an increased proliferation of Teffs is found in end stage animals (Beers et al., 2011a; Zhao et al., 2012). The innate immune system is also affected in ALS rodents, displayed by the substantial increase of NKT cells firstly in the liver and then in the spinal cord of *SOD1*^*G93A*^ mice (Chiu et al., 2008; Finkelstein et al., 2011).

Whether neuroinflammation is a cause or a consequence of motoneuron dysfunction is still debated. It is interesting to note that inflammation is not limited to the CNS but systemic with a correlation between disease evolution and levels of plasma LPS as well as the numbers of activated circulating monocytes and T lymphocytes (Zhang et al., 2005, 2009). A thymic dysfunction also parallels the neurodegenerative process in mutant *SOD1* mice and ALS patients (Seksenyan et al., 2010). In the CNS of ALS patients, TAR DNA-binding protein 43 (TDP-43) displays an increased expression and interacts with nuclear factor kappa B (NF-κB) in glial and neuronal cells. LPS-activation of NF-κB in microglial cells expressing the TDP-43 mutant is associated with the production of pro-inflammatory cytokines, including TNFα, IL-1β, IL-6, and IFNγ (Swarup et al., 2011). NF-κB, is also an important intermediate of the TLR signaling pathway that contribute to the initiation of inflammatory responses (O’Connell et al., 2012). The central role of inflammation and NF-κB in ALS was recently confirmed by the description in familial ALS of mutations in the gene encoding optineurin, a negative regulator of TNF-induced NF-κB activation (Maruyama et al., 2010).

Additional regulators of the neuroinflammatory response are the microRNAs (miRNA), an abundant class of small, non-coding RNA that regulate gene expression in a wide range of biological processes (O’Connell et al., 2012). Recently, a dominantly inherited mutation in the heterogeneous nuclear ribonucleoprotein (hnRNP) A1 has been associated with familial ALS (Kim et al., 2013). hnRNPA1 is a RNA-binding protein involved in RNA metabolism, including the regulation of alternative pre-mRNA splicing, mRNA export, and stability as well as the processing of miRNA (Guil and Caceres, 2007). Interestingly, hnRNPA1 can directly interact with TDP-43 (Buratti et al., 2005), and TDP-43 was proposed to contribute to the post-translational processing of miRNA through interaction with the endonucleases, Drosha and Dicer (Kawahara and Mieda-Sato, 2012). The activity of Dicer, which processes miRNA precursors at the RNA-induced silencing complex (Wilson and Doudna, 2013), is required to maintain motoneuron functional integrity. Indeed, the conditional deletion of *Dicer* in vesicular acetylcholine transporter-expressing cells leads to motoneuron degeneration and denervation atrophy in mice (Haramati et al., 2010). Another intriguing link with the miRNA pathway in the neuro-immune interaction has been recently revealed by the demonstration that the neurotransmitter acetylcholine can inhibit the production of pro-inflammatory cytokines, TNFα and IL-6, through induction of miRNA-124 in macrophages (Sun et al., 2013). In addition, a subset of CD4^+^ T cells has been described to produce acetylcholine to modulate the inflammatory response taking part of the autonomic homeostatic reflexes (Rosas-Ballina et al., 2011). Regarding ALS pathogenesis, a dysfunction of the cholinergic circuit has been reported in the spinal cord of SOD1 mutant mice, early in the disease course (Casas et al., 2013). Moreover, the choline acetyltransferase mRNA is a target of TDP-43 (Polymenidou et al., 2011), and the decrease in cholinergic input in the neuroinflammatory context of Alzheimer’s disease was also shown to lead to the down regulation of hnRNPA1 (Berson et al., 2012). Despite the sequential events implicating miRNAs and the cholinergic signaling needs to be further explored, this evidence concurs toward the contribution of the neuro-immune interaction in the degenerative process.

The information from pre-clinical models and ALS patients suggests that systemic immune activation (innate and adaptive) might play a key role in ALS pathogenesis and may represent an interesting target for the development of novel treatments. However, a better understanding of the specific roles played by the different subtypes of immune cells is of utmost necessity. Indeed, accumulative evidence suggests that inflammatory cells mediate both protective and deleterious effects on motoneuron survival and that these functions vary during disease progression.

### THE PROTECTIVE FUNCTION OF THE IMMUNE RESPONSE IN ALS

Protective immunity, a crucial homeostatic phenomenon in the repair of damaged tissues, results from both the clearance of debris and the effects of cytokines and growth factors delivered by inflammatory cells to the site of injury (Hohlfeld et al., 2000; Schwartz and Moalem, 2001). The neuroprotective ability of immune cells is also evident in ALS. Indeed, when *SOD1*^*G93A*^ mice are bred with mice lacking functional T cells or CD4^+^ T cells, microglia skew toward an M1 inflammatory phenotype and disease progression accelerates, suggesting that CD4^+^ T cells provide neuroprotection by suppressing the activation of cytotoxic microglia. Accordingly, reconstitution of T cells following bone marrow transplantation of *SOD1*^*G93A*^ mice lacking functional T and B cells prolonged their survival and suppressed the activation of M1 microglia (Beers et al., 2008). Further analysis showed that neuroprotection is mainly supported by CD4^+^CD25^+^Foxp3^+^ Tregs that secrete IL-4, thus promoting M2 protective microglia and IL-4 secreting Th2 cells, while inhibiting the neurotoxic Th1 response and IFNγ secretion. The passive transfer of Tregs into ALS mice lacking functional T cells results in lengthened disease duration and prolonged survival (Beers et al., 2011a). Accordingly, these neuroprotective Tregs are increased in the peripheral blood of ALS patients during early stages but their numbers decrease as the disease progression accelerates and are thus inversely correlated with disease progression rates (Beers et al., 2011a; Rentzos et al., 2012; Henkel et al., 2013). Furthermore, Foxp3 and CD25 expression is reduced in Tregs from rapidly progressing patients and are also inversely correlated with disease progression rates (Henkel et al., 2013). Co-culture experiments showed that Tregs suppress the expression of cytotoxic factors Nox2 and iNOS from *SOD1*^*G93A*^ microglia through IL-4 secretion and inhibit the proliferation of *SOD1*^*G93A*^ Teffs via the combined secretion of IL-4, IL-10, and TGF-β (Zhao et al., 2012). Hence, Tregs enhance the neuroprotective properties of the immune system during the stable disease phase while a switch from a neuroprotective Tregs/M2 to a deleterious Th1/M1 response characterizes disease progression. The key role of this balance between protective and deleterious immune responses in modulating clinical outcome is confirmed by the temporal and regional association between neuroinflammation and motoneuron injury in ALS mice (Beers et al., 2011b). Indeed, initial weakness in the hindlimbs is associated with a Th1 proinflammatory infiltrate in the lumbar spinal cord, while a protective Th2 immune response is observed in the cervical cord and may explain the delayed motor weakness in the forelimbs (Beers et al., 2011b). Therefore, the inflammatory infiltrate observed in ALS lesions appears not simply as a consequence of motoneuron degeneration but is actively involved in the neurodegenerative process. Tregs and Th2 lymphocytes assume the majority of the neuroprotective functions of the immune system and targeting their signaling pathways may be an attractive therapeutic strategy in ALS.

### THE NEUROTOXIC FUNCTION OF THE IMMUNE RESPONSE IN ALS

Cytotoxic T lymphocytes and NK cells are important effector cells of the immune system that eliminate aberrant cells, classically virus-infected cells, or tumorigenic cells (Zhang and Bevan, 2011; Kaur et al., 2012). Interestingly, at symptomatic stage, an increased number of CD8^+^ T and NK cells is observed in the blood and spinal cord of ALS patients (Calvo et al., 2010; Rentzos et al., 2012). Neurotoxic effects might be associated with a Th1-driven CTL pro-inflammatory immune response. Accordingly, mutant *SOD1* Th1 lymphocytes proliferate to a greater extend and produce more IFNγ during the rapidly progressing phase than Th1 lymphocytes isolated during the slowly progressing phase.

Different death pathways induced by CD8^+^ CTL lymphocytes could potentially lead to motoneuron death in ALS. CTL are antigen-specific effector cells that express the ligand for Fas (FasL) and most potential CTL targets express Fas at their surface. The activation of Fas (CD95) by its cognate ligand FasL commits cells to a death program through a caspase cascade (Peter et al., 2007). Interestingly, the activation of Fas triggers a death pathway in motoneurons that appeared restricted to this cell type (Raoul et al., 1999, 2002, 2006; Bernard-Marissal et al., 2012; Aebischer et al., 2013). Motoneurons expressing ALS-linked SOD1 mutations showed an increased susceptibility to Fas-mediated death through activation of a Fas/NO amplification loop (Raoul et al., 2002, 2006). Accordingly, mutant *SOD1* mice with homozygous loss-of-function FasL mutation present a reduced loss of motoneurons and a prolonged life expectancy (Petri et al., 2006). It remains to be determined whether CTL contribute to Fas-induced motoneuron loss. Another cytotoxic mechanism of CTL-mediated killing of target cells is the perforin-granzyme system. Upon recognition of a target cell by CTL, cytotoxic granules containing perforin and granzyme are released in the extracellular space. Perforin is a pore forming protein allowing the entry in the target cells of granzyme serine proteases that subsequently induce caspase activation and cell death (van Domselaar and Bovenschen, 2011). It is noteworthy that increased levels of granzyme A and B isoforms are increased in the serum of ALS patients (Ilzecka, 2011). However, the functional significance of such an increase remains to be determined. IFNγ, which is produced by CTL cells, can exert both immunostimulatory and immunomodulatory effects during an immune response. IFNγ produced by mutant astrocytes and motoneurons can elicit a death program in motoneurons through the activation of the lymphotoxin beta receptor (LT-βR) by its ligand LIGHT (Aebischer et al., 2011, 2012). The genetic deletion of *Light* in *SOD1*^*G93A*^ mice suggests that the LIGHT pathway contributes to the progression phase of the disease. Recently, the intracerebroventicular infusion of neutralizing anti-IFNγ antibody has been shown to delay the motor function decline in *SOD1*^*G93A*^ mice, suggesting that IFNγ contributes to ALS pathogenesis (Otsmane et al., 2013). However, The precise contribution of IFNγ in the neuroinflammatory response remains to be investigated.

An infiltration of NK cells has been reported in the spinal cord of symptomatic ALS mice (Chiu et al., 2008). While the role of NK cells in ALS remains unknown, several hypothetical mechanisms can be raised about their pathogenic contribution. Indeed, activated NK cells inhibit neurite outgrowth of cerebellar neurons in a cell contact-dependent manner *in vitro* (Pool et al., 2012). In sensory neurons, IL-2-activated NK cells have a killing activity that requires the perforin-granzyme system (Backstrom et al., 2000). Further, the production of IFNγ by activated NK cells might directly trigger motoneuron death through the LIGHT/LT-βR pathway or potentiate a cytotoxic Th1/CTL response via the combined action of other NK-related cytokines such as IL-17 or IL-22 (Cella et al., 2010). NK cells thus represent an interesting branch of the immunopathology that should be further considered.

**FIGURE 1:**
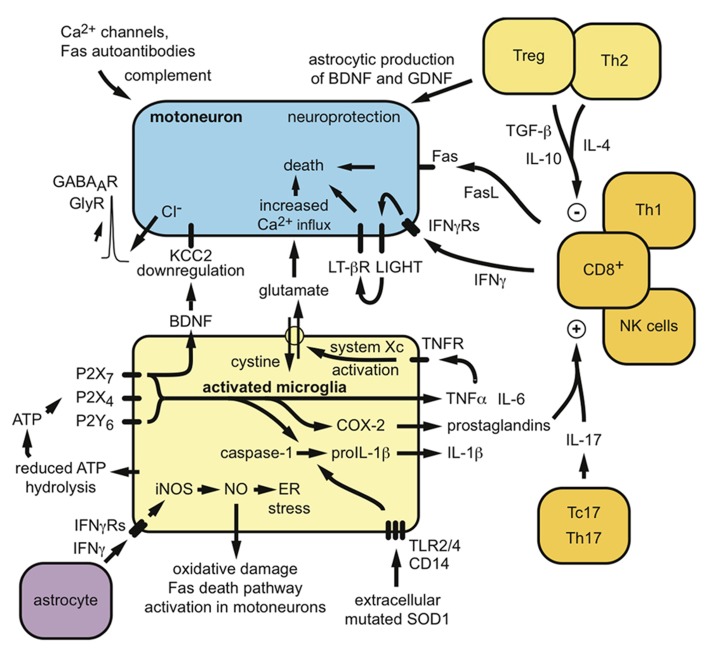
**Potential mechanisms by which peripheral and central immunity might contribute to the neurodegenerative process in ALS.** Both neuroprotective and neurotoxic functions can be proposed for the involvement of microglia and lymphocytes in ALS pathogenesis.

Several studies suggest that humoral immunity and immunoglobulins could also contribute to the disease. Autoantibodies to voltage-gated Ca^2^^+^ or K^+^ channels have been described in ALS patients, which induce specific motoneuron alterations both *in vitro* and *in vivo* after passive transfer in mice (Appel et al., 1991; Engelhardt et al., 1995; Demestre et al., 2005; Pagani et al., 2006; Nwosu et al., 2010). Abnormal levels of anti-Fas antibodies, able to induce neuronal apoptosis *in vitro*, have been detected in the serum of patients with ALS (Yi et al., 2000; Sengun and Appel, 2003). C5a and other complement activation products released after activation of the classical complement pathway by antibodies are elevated in the CSF and spinal cord of ALS mice and patients and specific inhibition of C5a receptor ameliorates disease in *SOD1*^*G93A*^ mice (Woodruff et al., 2008; Heurich et al., 2011). Thus, both the innate and adaptive immune system appears to have deleterious consequences on the survival and maintenance of motoneurons in ALS (**Figure 1**).

## EXPLOITING THE NEUROPROTECTIVE AND NEUROTOXIC PROPERTIES OF NEUROIMMUNITY FOR THE DEVELOPMENT OF THERAPEUTIC STRATEGIES

In light of the dynamic functional changes of microglia and immune cells discussed above, attempts to develop therapeutic strategies targeting neuroinflammation have only emphasized the importance of understanding the temporal neuroinflammatory events in ALS.

In pre-clinical mouse models, genetic deletion of the P2X_7_ receptor, which was previously described as being upregulated in ALS microglia (D’Ambrosi et al., 2009), resulted in increased motoneuron loss, increased microgliosis, and accelerated disease progression, thus suggesting an unanticipated protective role for the P2X_7_ receptor (Apolloni et al., 2013). Similarly, as mentioned earlier, genetic depletion of functional T cells or CD4^+^ cells in *SOD1*^*G93A*^ mice lead to increased disease progression, decreased survival as well as promoted production of pro-inflammatory effectors (Beers et al., 2008). Finally, eliminating the expression of galectin-3, a multifunctional immunomodulator that is increased in ALS microglia (Norling et al., 2009), in *SOD1*^*G93A*^ mice, also results in aberrant microgliosis and increased disease progression (Lerman et al., 2012). These alterations (P2X_7_, immune cells and galectin-3) were embryonically and permanently induced, implying that at a certain time-point during the development of the animal and the progression of the disease, these molecular and cellular components are necessary for alleviating certain ALS symptoms and pathological features.

At the clinical level, the failure of certain trials assessing the influence of drugs that directly or indirectly impact neuroinflammation may be due to inappropriate knowledge of the dynamic changes that occur within microglia and immune cells. Indeed, drastic immunosuppressive strategies such as cyclosporine, cyclophosphamide, intravenous immunoglobulin G treatment, and total lymphoid irradiation did not provide any significant benefits to ALS patients (Brown et al., 1986; Drachman et al., 1994; Gourie-Devi et al., 1997). Similarly, drugs used to target specific neuroinflammatory effectors that showed promising results in pre-clinical models such as celecoxib and pioglitazone (Drachman et al., 2002; Schutz et al., 2005), proved to be ineffective in improving motor functions and survival in ALS patients (Cudkowicz et al., 2006; Dupuis et al., 2012).

The progressive spreading, extension and diffusion of the neurodegenerative process that typically occurs in ALS patients may result from the concurrent progressive invasion of the CNS by glial cells and most importantly, the functional changes that take place within these cells. Importantly, an incomplete understanding of said changes could lead to undesired and unexpected results. Indeed, both minocycline and thalidomide (an analog of lenalidomide) revealed serious harmful effects in patients during a randomized placebo-controlled phase III trial and a single arm, open label phase II study, respectively (Gordon et al., 2007; Stommel et al., 2009).

As translational therapy targeting neuroinflammatory and immunomodulatory effectors is rapidly progressing, it has become clear that a step backward is presently required to better assess the temporal functional changes that occur within glial and immune cells in ALS pathogenesis. The cellular environment being composed of both neuroprotective and neurotoxic functions, specific therapeutic windows may dictate the choice of drugs and their pathogenic targets. Alternatively, a combinatory therapeutic approach may be more efficient at modulating the contributions of non-neuronal cells to ALS pathology. Thus, while neuroinflammation undoubtedly plays a role in ALS pathogenesis, therapeutic success will be reached in limiting the activation and amplification of toxic glial and immune cells whilst preserving the cellular subtypes that are beneficial to motoneuron survival.

## Conflict of Interest Statement

The authors declare that the research was conducted in the absence of any commercial or financial relationships that could be construed as a potential conflict of interest.
